# The relationship between COVID-19 anxiety and self-efficacy among adolescent students: A cross-sectional study

**DOI:** 10.1371/journal.pone.0310434

**Published:** 2024-12-05

**Authors:** Parvin Mangolian Shahrbabaki, Somayeh Zeidabadinejad, Parniya Abolghaseminejad, Mahlagha Dehghan, Marzieh Asadilari, Mohammad Ali Zakeri, Ghada Shahrour, Leyla Ahmadi Lari

**Affiliations:** 1 Nursing Research Center, Kerman University of Medical Sciences, Kerman, Iran; 2 M.Sc in Critical Care Nursing, Nursing Research Center, Sirjan University of Medical Sciences, Sirjan, Iran; 3 MSc in Health Education, Sirjan School of Medical Sciences, Sirjan, Iran; 4 Department of Nursing, M.Sc Nursing, School of Nursing, Larestan University of Medical Sciences, Larestan, Iran; 5 MSc in Nursing, Clinical Research Development Unit, Ali-Ibn Abi-Talib Hospital, Rafsanjan University of Medical Sciences, Rafsanjan, Iran; 6 Faculty of Nursing, Jordan University of Science and Technology, Irbid, Jordan; 7 College of Nursing, RAK Medical and Health Sciences University, Ras Al-Khaimah, UAE; 8 Department of Anesthesiology, M.Sc in Critical Care Nursing, School of Nursing, Larestan University of Medical Sciences, Larestan, Iran; 9 Hazrat Zeinab Faculty of Nursing and Midwifery, Larestan University of Medical Sciences, Dr. Dadman Highway, Larestan, Iran; Tehran University of Medical Sciences, ISLAMIC REPUBLIC OF IRAN

## Abstract

**Background:**

The spread of the COVID-19 epidemic, as well as its high contagiousness, increased students’ anxiety and stress. Anxiety may affect individuals’ functioning and undermine their self-efficacy. Therefore, the present study aimed to determine the relationship between COVID-19 anxiety and self-efficacy among adolescent students.

**Method:**

This cross-sectional descriptive correlational study was conducted on 306 adolescent students in southern Iran. The research tools were the Corona Disease Anxiety Scale consisting of physical and psychological dimensions of anxiety and the Self-Efficacy Scale composed of three subscales: academic, social, and emotional self-efficacy. Data were analyzed using SPSS 25. Pearson correlation was used to investigate the relationship between COVID-19 anxiety and self-efficacy using the total score of both scales and their dimensions as well. T-Test and one-way ANOVA were used to see whether sociodemographic variables of participants significantly varied in self-efficacy and COVID-19 anxiety.

**Results:**

The mean score of COVID-19 anxiety was 11.70 ± 9.25, representing mild anxiety, and the mean score of the psychological dimension was higher than that of the physical dimension. The mean self-efficacy score was 81.29 ± 12.98 indicating high level of self-efficacy. In addition, the mean score of academic self-efficacy was higher than that of social and emotional self-efficacy. There was an inverse, significant, and mild to moderate relationship between COVID-19 anxiety and the total score of self-efficacy (*r* = -.28, *p* < .001) and all its dimensions (*r* = -. 18, *p* = .002 for social and academic self-efficacy; *r* = -.32, *p* <. 001 for emotional self-efficacy). While the physical dimension of COVID-19 anxiety correlated significantly and negatively with self-efficacy total and subscale scores, the mental subscale of COVID-19 anxiety only correlated with the self-efficacy total score (*r* = -.20, *p* < .001) and emotional self-efficacy (*r* = -.28, *p* < .001).

**Conclusion:**

The study results revealed that the higher the COVID-19 anxiety, the lower the self-efficacy among adolescents. It is important to prioritize the enhancement of self-efficacy among adolescents to ensure their success across various life domains including managing stressors brought about by pandemics like COVID-19. This requires a collaborative effort from parents and teachers as they are a primary source of reassurance and information. Parents and teachers can also serve as role models in showcasing resilience and positive coping with the pandemic and allow adolescent students to master adaptive coping and provide positive reinforcement for effective behaviors.

## Introduction

The dissemination of infectious viral diseases, notably COVID-19, represents a substantial threat to public health. COVID-19, an acute respiratory illness, swiftly escalated into a global health crisis due to its virulence and rapid transmission [1]. As of July 13, 2022, the virus had infected over 500 million individuals worldwide, resulting in more than 6 million fatalities. In Iran alone, 7,376,794 cases and 141,891 deaths were documented, positioning the country as 17th globally in terms of COVID-19-related mortality [2].

The repercussions of COVID-19 have been profound, exacerbating morbidity and mortality rates, and exerting pressure on economic stability and healthcare infrastructures. Vulnerable demographics, including the elderly, individuals with pre-existing conditions, and minorities, experienced disproportionately adverse health outcomes and elevated mortality rates [3]. Healthcare systems confronted severe challenges, such as shortages in critical care resources, limited access to ventilators, and an overburdened workforce [4]. Moreover, the pandemic precipitated various physical and psychological health issues, including cognitive impairments, cardiovascular and respiratory problems, persistent fatigue, and social anxiety [5].

Anxiety, a generalized response elicited by diverse stressors, disrupts biological, physical, and psychological homeostasis [6]. Elevated stress and anxiety levels can compromise the immune system, thereby increasing susceptibility to illnesses such as COVID-19 [7]. Numerous countries instituted restrictive measures to mitigate the spread of the virus, engendering fears of illness and death, the propagation of misinformation, disruption of daily routines, reduced social interactions, and occupational and financial difficulties. Collectively, these factors imposed a substantial psychological burden on families and students, detrimentally impacting mental health [8,9]. The mental health of students, in particular, deteriorated following school closures or transitions to virtual learning environments, with anxiety levels surging [10]. Anxiety during adolescence can precipitate depression and impair academic performance [11]. It has enduring negative ramifications on students’ lives, with severe anxiety potentially disrupting daily functioning [12].

Anxiety diminishes performance levels, and self-efficacy plays a pivotal role in performance [12]. Bandura’s theory defines self-efficacy as an individual’s belief in their capacity to execute self-care effectively and attain desired outcomes [13]. Bandura asserts that physiological and emotional arousal influences self-efficacy, with heightened arousal correlating with diminished self-efficacy [14]. Self-efficacy is multifaceted, encompassing social, emotional, and academic dimensions. Social self-efficacy facilitates the formation of social connections and the management of environmental pressures [15], emotional self-efficacy pertains to the regulation of feelings and emotions during social interactions [16], and academic self-efficacy relates to students’ confidence in their ability to comprehend and complete academic tasks [17]. Individuals with high self-efficacy exhibit greater motivation, better health outcomes, enhanced success, and improved social integration. They possess metacognitive skills essential for the effective completion of tasks [16,17]. Conversely, individuals with low self-efficacy harbor doubts about their abilities, exhibit low motivation, and demonstrate slower task completion rates, with low social and academic self-efficacy levels predicting long-term depression in students [18,19]. Research has identified significant inverse relationships between anxiety and self-efficacy. For example, Spanish students with elevated anxiety levels exhibited negative emotions and low academic self-efficacy [18]. Studies on emotional intelligence and perceived self-efficacy similarly revealed an inverse correlation between perceived self-efficacy and stress, with high stress levels associated with low self-efficacy [19]. Additionally, the COVID-19 pandemic negatively impacted students’ self-efficacy, with increased anxiety leading to reduced self-efficacy [20].

Students are susceptible to environmental hazards, and their future physical and mental health is profoundly influenced by their childhood experiences. It is imperative to investigate the psychological impact of the COVID-19 epidemic on this demographic, as their mental health is crucial for the overall well-being of families and society. In light of the paucity of similar studies, this research aims to examine the relationship between COVID-19 anxiety and self-efficacy among adolescent school students in 2021, with the objective of identifying individuals at risk of psychological disorders under high-risk conditions such as the COVID-19 pandemic.

## Materials and methods

### Study design and setting

This cross-sectional descriptive correlational study was conducted on male and female adolescent students in southern Iran from October to mid-December 2021. A correlational study is suitable since the aim of this study was to investigate the relationship between adolescent students’ COVID-19 anxiety and their self-efficacy.

### Study sample

This study was performed on primary and secondary schools in southeastern Iran. Schools were conveniently selected from each district, then the list of students was taken from the school principal and they were contacted using a table of random numbers. G power calculation was used to estimate the required sample size using the following parameters for Pearson correlation analysis: small to medium effect size of 0.2, 95% level of confidence, and 90% test power, yielding a total sample size of 314. In this study, a total of 320 students completed the study questionnaire. Inclusion criteria required students to be in good mental and psychological health and not to have lost any close relatives within the previous six months. Adolescents with a history of known psychological diseases such as psychosis, depression, personality disorder, separation anxiety, panic disorder and others were excluded from the study. This exclusion occured after the data collection was completed by removing those questionnaires where the corresponding students indicated they had a mental disorder.

#### Instruments

This study used demographic and background information questionnaire, the Corona Disease Anxiety Scale and self-efficacy scale.

Demographic and background information questionnaire included age, education level, parents’ occupation, parents’ education level and number of children, having a specific mental illness and existing chronic physical disease.

### The Corona Disease Anxiety Scale (CDAS)

The CDAS was prepared and validated by Alipour et al. to measure anxiety caused by the spread of the Corona virus in Iran [21]. This tool consists of 18 items divided into two subscales. Items 1 to 9 measure psychological symptoms and items 10 to18 measure physical symptoms. This measure is scored on a four-point scale. Therefore, the highest and lowest scores are between 0 and 54 with higher scores indicating a higher level of anxiety in individuals. The total score of anxiety severity is divided into three categories: mild (0–16), moderate (17–29), and severe (30–54). The Guttman’s λ 2 value for the whole questionnaire was obtained as (λ = 0.922), Cronbach’s alpha coefficient for psychological symptoms as (α = 0.879), physical symptoms as (α = 0.861), and for the whole questionnaire as (α = 0.919) (31). In the study by Mangolian et al, the content validity index was determined to be 0.91 by faculty members. Cronbach’s alpha coefficient was calculated to be 0.99 through the completion of this questionnaire by participants to confirm the internal consistency of this measure [22].

### Self-efficacy scale

Muris (2001) developed self-efficacy scale [23] to evaluate the self-efficacy of school-aged children and adolescents (7–18 years old), with three sub-scales of social, academic and emotional self-efficacy. This 23-item questionnaire measures the subject’s ability in different situations. Each item is rated on a 5-point Likert scale (from 1 = not at all to 5 = very much). The subscales and items related to this questionnaire include social self-efficacy (items 1–8), academic self-efficacy (items 9–16), and emotional self-efficacy (items 17–23), with a higher score reflecting higher self-efficacy. The range of scores for general self-efficacy, social and academic self-efficacy, and emotional self-efficacy is 23–115, 8–40, and 7–35, respectively. Morris (2001), in addition to examining the convergent and divergent validity of the scale, reported total reliability of the scale (0.70), social self-efficacy (0.78), emotional self-efficacy (0.80), and academic self-efficacy (0.87). Tahmasian (2007) confirmed the reliability and validity of this tool in Iran and reported test-retest reliability for the whole scale (0.89), social self-efficacy (0.81), emotional self-efficacy (0.88), and academic self-efficacy (0.87) [20].

### Data collection

Sampling began after the approval of the proposal, acquisition of the code of ethics, provision of the introduction letter to the schools, and coordination with the authorities. As the students were quarantined at home and it was impossible to collect information in person, the online questionnaires were distributed in virtual groups. Since the students might not be in good physical and mental health due to the conditions of COVID-19 and quarantine, they were asked to complete the questionnaire whenever they could and had enough time to answer the questions. For this purpose, every two days, a reminder message was sent in virtual groups. Students’ assent was included in the first page of the questionnaire as they were asked to click the agree button if they were willing to take part in the study. Parents of the randomly selected students were contacted virtually and those who agreed to have their child to participate in the study, their children were added in the virtual groups. Data collection began in October 2021 and ended in December of 2021. Institutional Review board approval was obtained from the Ethics Committee of Kerman University of Medical Sciences (IR.KMU.REC.1400.373).

### Data analysis

SPSS-25 was used for data analysis. Descriptive statistics (frequency, percentage, mean and standard deviation) were used to describe the background characteristics of study participants as well as to determine the COVID-19 anxiety and self-efficacy scores. We also ran the normality tests separately for the study variables (i.e., COVID-19 anxiety and self-efficacy) and the results showed that the Kolmogorov-Smirnov test and the Shapiro wilk value were both non-significant (p > .05), indicating normal distribution of both variables. Furthermore, the Q-Q plot confirmed the normal distribution of COVID-19 anxiety and self-efficacy. Therefore, Pearson correlation was used to investigate the relationship between COVID-19 anxiety and self-efficacy. Furthermore, independent t and ANOVA tests were used to determine the relationship between the COVID-19 anxiety score and the demographic characteristics of the students and whether self-efficacy also varied according to sociodemographic characteristics of the participants. A significance level of < 0.05 was considered.

## Results

According to the results, of the 320 eligible participants, 306 students were finally analyzed, of which 216 (70.6%) were boys and 90 (29.4%) were girls. The sample mean age was 14 ± 1.76 years. More than one third of the students (35.6%) were in 10th grade. Parents’ education was mostly limited to a high school diploma (30.7% for fathers and 35.9% for mothers) or less (30.4% and 22.9% for fathers and mothers, respectively). Regarding parents’ employment, the majority of father were self-employed (52.9%), while 72.1% of mothers were unemployed. The majority of the participants had no history of mental disorders (98.4%) or chronic illness (94.7%) (Table 1).

**Table 1:** Participant’s characteristics and descriptives of study variables.

Characteristic	Subgroup	N (%)
Sex		
	Male	216 (70.6)
	Female	90 (29.4)
Age (years)	10–12	47 (15.3)
	13–14	84 (27.5)
	15	66 (21.6)
	16	109 (35.6)
Grade		
	4–6	47 (15.3)
	7–8	84 (27.5)
	9	66 (21.6)
	10	109 (35.6)
Father education		
	< diploma	93 (30.4)
	Diploma	94 (30.7)
	Associate Degree	22 (7.2)
	BS.C	58 (19.0)
	M.SC or higher	39 (12.7)
Mother education		
	< diploma	70 (22.9)
	Diploma	110 (35.9)
	Associate Degree	23 (7.5)
	BS.C.	75 (24.5)
	M.SC or higher	28 (9.1)
Father job		
	Unemployed or retired	40 (13.1)
	Employee	86 (28.1)
	self-employment	162 (52.9)
	private job	18 (5.9)
Mother job		
	Unemployed	218 (71.2)
	Retired	7 (2.3)
	Employee	63 (20.6)
	self-employment	12 (3.9)
History of mental illness		
	Yes	5 (1.6)
	No	315 (98.4)
History of chronic illness		
	Yes	17 (5.3)
	No	303 (94.7)
**Variable**	**M**	**SD**
**COVID-19 anxiety**	11.70	9.25
Mental	9.06	6.27
Physical	2.64	3.83
**Self-efficacy**	81.29	12.98
Social	28.20	5.17
Educational	30.40	5.57
Emotional	22.69	5.57

M: Mean, SD: Standard deviation.

According to the Corona Disease Anxiety Scale, the item“I am worried about the spread of Coronavirus disease to the people around me,” with a mean of 1.75 (*SD* = 1.06) caused the most anxiety among participants, followed by the items “I am very worried about the spread of the coronavirus disease,” and “I am afraid of becoming infected with the coronavirus”(*M* = 1.27, *SD* = .98 and *M* = 1.26, *SD* = 0.99, respectively). Furthermore, “I get a headache when I think about Coronavirus disease” (*M* = 0.16, *SD* = 0.40) and “Thinking about Coronavirus disease has made me lose my appetite” (M = 0.15, SD = 0.42) caused the least amount of anxiety.

The mean score of COVID-19 anxiety was 11.70 ± 9.25 (mild). In addition, the mean score of the mental dimension (9.06± 6.27) was higher than that of the physical dimension (2.64± 3.83). The CDAS scale of anxiety is divided into three categories in terms of severity: mild (0–16), moderate (17–29), and severe (30–54). The majority of participants (71.3%) experienced mild anxiety, while (19.1%) reported moderate anxiety and 9.6% reported severe anxiety. The mean score of self-efficacy was 81.29 ± 12.98 (a high level). Furthermore, the mean score of academic self-efficacy (30.40± 5.57) was higher than that of social (28.20± 5.17) and emotional (22.69± 5.57) self-efficacy (Table 1).

**Fig 1:**
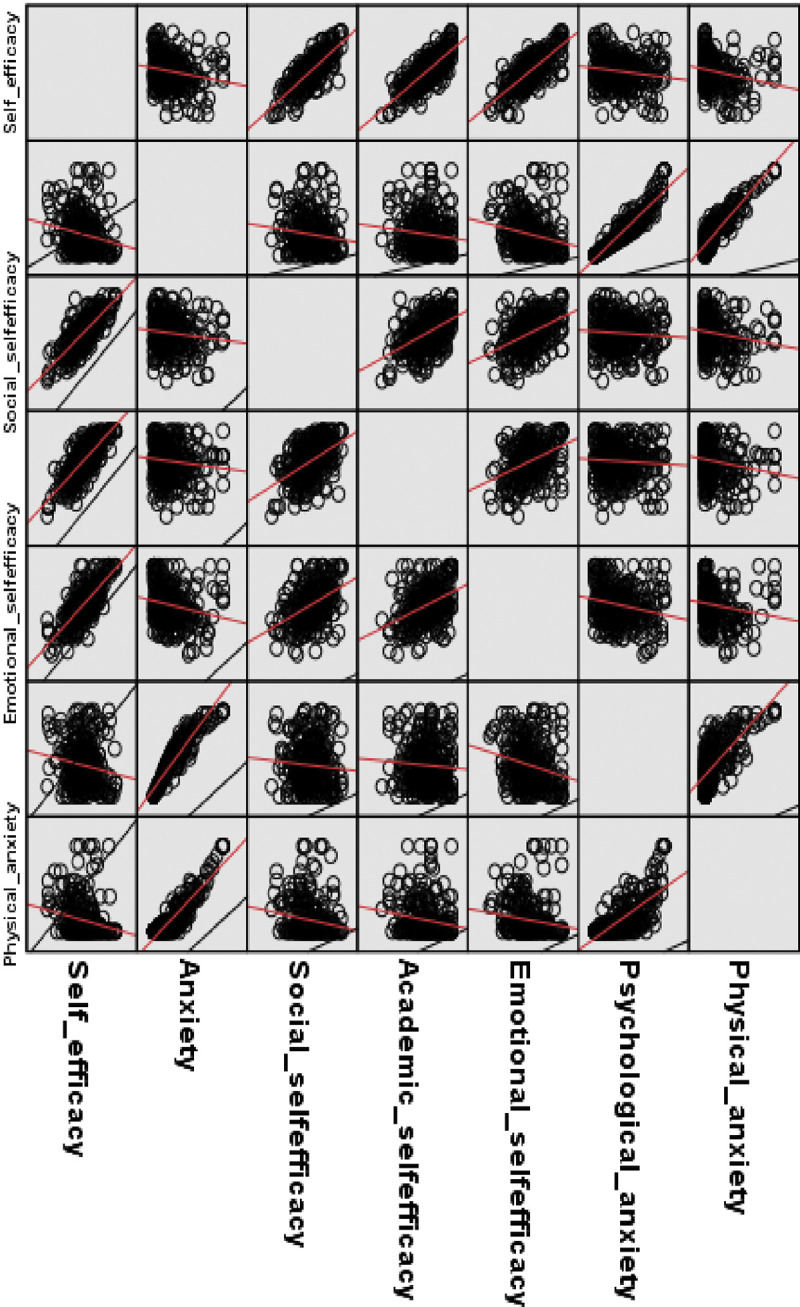
Scatter plot of the relationship between COVID-19 anxiety and self-efficacy. This figure displays the linear relationship between COVID-19 anxiety and self-efficacy as total scales and subscales using Pearson correlation coefficient.

Regarding the relationship between COVID-19 anxiety and self-efficacy, there was a statistically significant, inverse, and mild to moderate relationship between COVID-19 anxiety total score and self-efficacy total score (r = -.28, p < .001) and a significant moderate relationship with the emotional subscale of self-efficacy (r = -.32, p < 0.001), in addition to a weak, inverse, and statistically significant relationship with academic and social self-efficacy (r = -.18, p = .002 each). The mental dimension of COVID-19 anxiety had a mild to moderate negative relationship with emotional self-efficacy (r = -.28, p < .001) and the total self-efficacy scale (r = -0.20, p <0.001), while the physical self-efficacy correlated negatively and inversely with the total score of COVID-19 anxiety (r = —.36, p < .001) and its dimensions; social self-efficacy (r = -.26, p < .001), academic self-efficacy (r = -.28, p < .001) and emotional self-efficacy (r = -.32, p < .001). No statistically significant relationship was observed between COVID-19 anxiety and the other self-efficacy domains (p > .05) (Table 2 and **Fig 1).**

**Table 2:** The correlation between COVID-19 anxiety and self-efficacy using pearson correlation analysis.

Variable	COVID-19 Anxiety					
	Mental		Physical		Total	
	r	P value	r	P value	r	P value
Self-efficacy	-0.20	**< 0.001**	-0.36	**< 0.001**	-0.28	**< 0.001**
social	-0.10	0.09	-0.26	**< 0.001**	-0.18	**0.002**
educational	-0.09	0.11	-0.28	**< 0.001**	-0.18	**0.002**
emotional	-0.28	**< 0.001**	-0.32	**< 0.001**	-0.32	**< 0.001**

Female students were significantly more anxious about COVID-19 than their male counterparts (t = -3.11, *p* = 0.002). In addition, students with pre-existing chronic illness had higher levels of anxiety than those without such a history (*t* = t = 3.25, *p* < .001). Other background variables were not significantly associated with coronavirus disease anxiety. The self-efficacy of male students was significantly higher than that of female students (*t* = 3.09, *p* = 0.002). There was no significant relationship between self-efficacy and other background variables.

## Discussion

This study addressed the relationship between self-efficacy and COVID-19 anxiety in students. The results showed that adolescent students reported mild anxiety level, but they scored high on the self-efficacy scale. The findings also revealed a significant inverse relationship between self-efficacy (in social, academic and emotional domains) and COVID-19 anxiety (in the physical and mental dimensions). In other words, the higher the COVID-19 anxiety, the lower the self-efficacy, and vice versa.

Adolescent students in our study reported mild level of COVID-19 anxiety, with a higher mean score on the mental dimension compared to the physical dimension. These findings are consistent with a study conducted among Iranian adolescents during the pandemic, which also reported mild COVID-19 anxiety, with greater anxiety observed in the mental rather than the physical dimension [24]. However, our findings were incongruent with another study of 300 Iranian adolescents who reported severe anxiety related to COVID-19 [25]. The latter study included adolescents who had family members diagnosed with COVID-19 and who had undergone laboratory testing for COVID-19 screening between 2019 and 2022, which may account for the elevated anxiety levels observed in that cohort.

In terms of self-efficacy, participants in our study demonstrated high levels, with academic self-efficacy scoring the highest compared to both emotional and social self-efficacy. Similarly, high self-efficacy was reported in an Iranian study investigating the mediating role of self-efficacy in the relationship between risk perception and psychological vulnerability among youth with social anxiety disorder [26]. However, when examining the components of self-efficacy, mixed results were found in the literature. While academic self-efficacy surpassed emotional and social dimensions in a study involving Iranian adolescents aged 10 to 18 [27], these findings contrast with a Malaysian study, which reported that academic self-efficacy was the lowest among adolescent students [28].

Many studies investigated the relationship between self-efficacy, general anxiety [29], exam anxiety [30], research anxiety [31], and speech anxiety [32]. Research also examined the relationship between self-efficacy and COVID-19 anxiety among students in Iran [33], and Spain [18], as well as among hospital employees in Iran [34] and Poland [35]. The results of the present study are consistent with these studies. For example, a moderate and statistically negative relationship was found between research self-efficacy and research anxiety (r = .32, p < .01), and self-efficacy negatively predicted research anxiety among graduate students [31]. Among 109 Iranian teachers, speech anxiety decreased as teachers’ self-efficacy increased (r = -.22, p < .01) [32]. In the context of COVID-19, self-efficacy was negatively correlated with COVID-19 anxiety (r = -.28, p < .01) and mediated the relationship between coping and COVID-19 anxiety among undergraduate Iranian students [33]. Among healthcare workers in Poland, those with lower levels of general self-efficacy, had higher tendency to experience COVID-19 anxiety [35]. The results of Simorangkir et al.’s emphasized the importance of the role of self-efficacy in reducing anxiety and creating motivation during the COVID-19 pandemic [36], which is consistent with the results of the present study.

Barrows et al, in their research found how self-efficacy in students was able to moderate students’ anxiety, especially when they were facing exams. Student self-efficacy has a very important role in building self-confidence in completing various tasks given by the teacher, even in all subjects in school, and generating achievement motivation in those subjects [37]. Griggs et al in their study showed that students who self-reported higher math and science anxiety also reported less self-efficacy toward these subjects [38]. Academic self-efficacy showed powerful relationships with test anxiety in the secondary and high school students [39,40]. Although these studies were conducted at a time other than COVID-19, but since they emphasize the importance of the role of self-efficacy on anxiety, they are also in line with the present study.

Another result was that girls had significantly higher levels of anxiety than boys did, which is consistent with the results of Wang [41], Alemany-Arrebola et al [18], Math et al [42], and Bidzan et al [35]. Studies by Radwan et al. and Mouloud et al. showed that there was a significant difference in stress, anxiety, and depression scores between genders in students during the COVID-19 pandemic [43,44]. These variations may be influenced by a combination of biological, cultural, and environmental factors [45]. In addition, females have higher sensitivity in fear-related neural networks and have different conditioned skin conductance responses to stimuli than males [46]. Female students experience more anxiety than male students due to the fear of losing their academic achievements during COVID-19, as well as fear of the negative impact of COVID-19 on health [47]. Normal and moderate levels of anxiety motivate individuals to engage in healthier behaviors in response to COVID-19, such as increased risk tolerance and preventive practices during the pandemic [48–50]. In this analysis, there were observed differences between men and women regarding risk perception. Studies have shown that gender is an important predictor of risk perception levels, with female gender predicting increased risk perception [51,52]. For this reason, it is necessary to pay special attention to adolescents in stressful situations such as the spread of the corona virus, and the family should provide special support to girls. Devoto and Connor highlighted the sensitivity of adolescents and suggested that gender differences should be taken into account even when fighting the COVID-19 epidemic and providing health care [52,53], so programs and services should be proportional to the female sex.

According to this study, people with a history of chronic disease have a higher level of anxiety than others. According to the review of the literature, no study was found that measured the relationship between anxiety and history of chronic illness in students. But in other studies, the relationship between these two components has been mentioned. Özdin et al, Zhong et al and Salari et al.’s studies showed that during the outbreak of COVID-19, the level of anxiety increased in people with chronic diseases [54–56]. These individuals are more vulnerable to contract COVID-19 as their immune system is compromised. Chronic illness has also been identified as a crucial risk factor of mortality due to COVID-19 [54–56], which may explain their increased anxiety.

Although the long-term impact of COVID-19 on patients with a history of chronic illness is not yet known, it seems that these patients are more prone to mental problems due to social distancing, and fear of being infected with COVID-19. Global prevalence of mental disorders such as depression, anxiety and substance use had increased drastically due to COVID-19 pandemic, especially among young individuals [57,58]. These mental disorders can worsen preexisting anxiety and increase their likelihood of contracting COVID-19 due to an underlying chronic medical condition.

### Study limitations

This study had several limitations. Due to the spread of the corona virus and the closure of schools, it was not possible to collect data in person, so online self-reported questionnaires were used among the students of Lar city. For this reason, the results should be generalized with caution. In addition, variables, such as educational, social and economic status and other demographic variables of students were not studied, which might have affected the results. Furthermore, we did not exclude those students who might have had a COVID-19 infection prior to data collection, which might have influenced the results of this study. Similar studies should consider replicating the findings of this study considering the aforementioned limitations. Future research needs also to examine the long-term consequences of COVID-19 anxiety on students’ self-efficacy and other performance factors.

## Conclusion

The study results showed that there was a significant and inverse relationship between anxiety of COVID-19 and self-efficacy. The higher anxiety displayed because of the pandemic, the lower self-efficacy students displayed. Reducing COVID-19 anxiety and enhancing self-efficacy among adolescent students requires a collaborative effort from parents and teachers. Parents and teachers can serve as a primary source of reassurance and information, providing accurate information about COVID-19 pandemic in a safe environment.

Parents and teachers can serve as role models in showcasing resilience and positive coping with the pandemic and allow adolescent students to master adaptive coping and provide positive reinforcement for effective behaviors. They also can help these adolescents in navigating their uncertainties regarding COVID-19 pandemic. These approaches will contribute not only to anxiety reduction of COVID-19, but also to the development and reinforcement of adolescents’ self-efficacy in managing the uncertainties associated with the pandemic. These approaches should be specifically emphasized among adolescent girls and those with a history of chronic illnesses since they showed higher COVID-19 anxiety compared to their counterpart. Bandura social cognitive theory emphasizes the importance of modeling behavior and providing opportunities for mastery experiences to improve individuals’ self-efficacy [13].

## Supporting information

S1 File (XLSX)
