# Correction: The relationship between COVID-19 anxiety and self-efficacy among adolescent students: A cross-sectional study

**DOI:** 10.1371/journal.pone.0333182

**Published:** 2025-09-23

**Authors:** Parvin Mangolian Shahrbabaki, Somayeh Zeidabadinejad, Parniya Abolghaseminejad, Mahlagha Dehghan, Marzieh Asadilari, Mohammad Ali Zakeri, Ghada Shahrour, Leyla Ahmadi Lari

The authors are listed out of order. Please view the correct author order, affiliations, and citation here:

Leyla Ahmadi Lari^8,9^, Parvin Mangolian Shahrbabaki^1^, Somayeh Zeidabadinejad^2^, Parniya Abolghaseminejad^3^, Mahlagha Dehghan^1^, Marzieh Asadilari^4^, Mohammad Ali Zakeri^5^, Ghada ShahrourID^6,7*^

**1** Nursing Research Center, Kerman University of Medical Sciences, Kerman, Iran, **2** M.Sc in Critical Care Nursing, Nursing Research Center, Sirjan University of Medical Sciences, Sirjan, Iran, **3** MSc in Health Education, Sirjan School of Medical Sciences, Sirjan, Iran, **4** Department of Nursing, M.Sc Nursing, School of Nursing, Larestan University of Medical Sciences, Larestan, Iran, **5** MSc in Nursing, Clinical Research Development Unit, Ali-Ibn Abi-Talib Hospital, Rafsanjan University of Medical Sciences, Rafsanjan, Iran, **6** Faculty of Nursing, Jordan University of Science and Technology, Irbid, Jordan, **7** College of Nursing, RAK Medical and Health Sciences University, Ras Al-Khaimah, UAE, **8** Department of Anesthesiology, M.Sc in Critical Care Nursing, School of Nursing, Larestan University of Medical Sciences, Larestan, Iran, **9** Hazrat Zeinab Faculty of Nursing and Midwifery, Larestan University of Medical Sciences, Dr. Dadman Highway, Larestan, Iran

Lari LA, Shahrbabaki PM, Zeidabadinejad S, Abolghaseminejad P, Dehghan M, Asadilari M, et al. (2024) The relationship between COVID-19 anxiety and self-efficacy among adolescent students: A cross-sectional study. PLoS ONE 19(12): e0310434. https://doi.org/10.1371/journal.pone.0310434
